# Efficacy of 0.5% Levofloxacin and 5.0% Povidone-Iodine Eyedrops in Reducing Conjunctival Bacterial Flora: Metagenomic Analysis

**DOI:** 10.1155/2020/1780498

**Published:** 2020-04-17

**Authors:** Caixia Fan, Baoxia Yang, Yusen Huang

**Affiliations:** ^1^Weifang Medical University, Weifang 261053, China; ^2^State Key Laboratory Cultivation Base, Shandong Provincial Key Laboratory of Ophthalmology, Shandong Eye Institute, Shandong First Medical University and Shandong Academy of Medical Sciences, Qingdao 266071, China

## Abstract

Bacteria associated with postoperative endophthalmitis mostly originate from the normal bacterial flora of the patient's conjunctiva and eyelids, so the incidence of endophthalmitis may be reduced by eliminating the ocular and adnexal flora before surgery. We assessed the effectiveness of eyedrops of 0.5% levofloxacin and 5.0% povidone-iodine (PVI) in reducing conjunctival bacterial flora by metagenomic analysis. A total of 2.4 × 10^6^ high-quality sequencing reads were generated from 93 conjunctival samples obtained from 31 eyes scheduled for cataract surgery before prophylactic therapy (group 1), after administration of 0.5% levofloxacin eyedrops into the conjunctival sac 8 times before surgery (group 2), and at 3 minutes after instillation of 5.0% PVI solution in the conjunctival sac (group 3) followed by surgery irrigation. The alpha diversity and beta diversity results demonstrated that group 3 had the least richness and biodiversity. *Corynebacterium, Pseudomonas, Staphylococcus, Acinetobacter*, and *Streptococcus* were predominant in all samples. The relative abundance of these bacterial species was 30.94%, 27.48%, 5.26%, 4.55%, and 2.61% in group 1, 16.32%, 44.10%, 2.19%, 5.39%, and 0.97% in group 2, and 5.90%, 65.55%, 0.39%, 5.36%, and 0.10% in group 3, respectively. The most easily and difficultly eliminated were *Corynebacterium* and *Pseudomonas*, respectively. In conclusion, the metagenomic analysis using high-throughput sequencing provides a scientific way for evaluating the effectiveness of a disinfection method from the perspective of analyzing the composition and diversity of the conjunctival microbiome. Despite the use of preoperative antisepsis regimens, the ocular surface of patients receiving cataract surgery could not be rendered completely aseptic, indicating that more strict disinfection methods need to be adopted to reduce the risk for anterior chamber contamination and endophthalmitis after cataract surgery.

## 1. Introduction

Endophthalmitis is a devastating complication following intraocular surgical interventions, with a reported incidence of 0.028% after cataract surgery, 0.011% after pars plana vitrectomy, 0.108% after penetrating keratoplasty, and 0.02% after intravitreal injection of antivascular endothelial growth factor agents [1, 2]. Since bacteria associated with postoperative endophthalmitis mostly originate from the normal bacterial flora of the conjunctiva and eyelids of patients [3–6], the incidence of endophthalmitis may be reduced by eliminating the ocular and adnexal flora before the intraocular surgery. Povidone-iodine (PVI) solution and topical antibiotic agents are often administered for periocular skin and conjunctival antisepsis [7].

Antibacterial effectiveness can be accurately measured with the total number of bacterial flora available. However, most related reports just assessed cultures as positive or negative and failed to quantify viable bacteria. The diversity of the uncultured bacteria is also considerable [8]. Molecular approaches have helped us improve our understanding of microbial communities, including 16S rDNA high-throughput sequencing, which also facilitates the discovery of novel genera and species [9–11]. The purpose of the study described herein was to determine the effectiveness of 0.5% levofloxacin and 5.0% PVI eyedrops in reducing conjunctival bacterial diversity and abundance before cataract surgery by metagenomic analysis.

## 2. Materials and Methods

### 2.1. Sample Collection

Thirty-one hospitalized patients (31 eyes) scheduled for cataract surgery were included. None of them had a history of systemic disease, ocular surface disease, uveitis, glaucoma, retinal disease, ocular trauma/transplantation, or contact lens wearing or received topical administration of antibiotics, corticosteroids, or nonsteroidal anti-inflammatory agents within 6 months.

All eyes were administered with one drop of 0.5% levofloxacin eyedrops (Santen, Osaka, Japan) 8 times into the conjunctival sac from 3 hours ahead of cataract surgery by nurses. Mydrin-P eyedrops (1% cyclopentolate hydrochloride and 2.5% phenylephrine hydrochloride; Santen, Osaka, Japan) were used twice 1 hour before surgery to dilate the pupil. In the preoperative preparation room, all patients received standard periorbital disinfection with 10.0% PVI scrub on the eyelids and surrounding skin after topical administration of anesthetic eyedrops.

The patients were transferred into the operating room, and then the brow, upper and lower eyelids, eyelashes, and the adjacent forehead, nose, cheeks, and temporal orbital area were again scrubbed with 10.0% PVI. After the surgical area was draped in a sterile fashion, a sterile lid speculum was placed in the eye. A few drops of 5.0% PVI solution were instilled into the conjunctival sac 3 minutes before surgery, which was followed by conjunctival rinsing with balanced salt solution (Alcon Laboratories, Fort Worth, TX, USA).

Conjunctival specimens were obtained at 3 time points: baseline (before prophylactic therapy; group 1), after the eighth administration of levofloxacin eyedrops (group 2), and after the instillation of 5.0% PVI solution (group 3). Samples were collected from the upper and lower palpebral conjunctiva and caruncle and fornix conjunctiva under topical anesthesia using disposable sterile dry absorbent cotton swabs, placed in sterile tubes, and stored in a −80°C freezer until DNA extraction. Meanwhile, a disposable sterile dry absorbent cotton swab with a topical anesthetic was put in a sterile tube as a blank control.

### 2.2. DNA Extraction

DNA was extracted from samples using the MicroElute Genomic DNA Kit (D3096-01; Omega, Guangzhou, China) according to the manufacturer's instructions. Blank samples of unused swabs were also processed through DNA extraction for verification of no DNA. The total DNA of swabs was eluted in 20 ml of elution buffer (Omega D3096) and stored at −80°C until measurement by PCR (Lianchuan Biotech, Hangzhou, China).

### 2.3. PCR Amplification and 16S rDNA Sequencing

Using the total DNA from the 31 eyes as a template and the primer (319F 50-ACTCCTACGGGAGGCAGCAG-30; 806R 50-GGACTACHVGGGTWTCTAAT-30), we amplified the V3-V4 regions of the bacterial 16S rRNA. All reactions were carried out in 25 ml (total volume) of mixtures, containing 25 ng of genomic DNA extracts, 12.5 ml of PCR premix, 2.5 ml of each primer, and PCR grade water, in the Mastercycler gradient thermocycler (Eppendorf, Hamburg, Germany) under initial denaturation at 98°C for 30 seconds, 35 cycles of denaturation at 98°C for 10 seconds, annealing at 54°C/52°C for 30 seconds, extension at 72°C for 45 seconds, and final extension at 72°C for 10 minutes. The PCR products were confirmed with 2% agarose gel electrophoresis. Throughout the DNA extraction process, ultrapure water, instead of a sample solution, was used to exclude the false positive PCR results as negative controls. The PCR products were then normalized by the AxyPrep Mag PCR Normalizer (Axygen Biosciences, Union City, CA, USA), and thus the quantification step could be skipped despite the PCR volume submitted for sequencing. The amplicon pools were prepared for sequencing with AMPure XT beads (Beckman Coulter Genomics, Danvers, MA, USA). The size and quantity of the amplicon library were evaluated with the LabChip GX (PerkinElmer, Waltham, MA, USA) and the Library Quantification Kit for Illumina (Kapa Biosciences, Woburn, MA, USA), respectively. The PhiX Control Library (Illumina), combined with the amplicon library (expected at 30%), was sequenced on 300PE MiSeq runs using the standard Illumina sequencing primers.

### 2.4. Data Analyses

The sequence reads were preprocessed with removal of the primer sequence. As the reads were expected to overlap by approximately 90 bp, the assembly process of high-quality 300PEs were performed using FLASH [12], with no preliminary quality trimming. The sequence reads were assembled using QIIME (version 1.7.0-dev) [13]. According to the criteria for QIIME quality trimming, which included truncation of the sequence before three consecutive low-quality bases and reevaluation for length, no ambiguous base calls, and minimal sequence length of 150 bp after trimming, about 5% to 10% of the reads were filtered out. The filtered sequences were clustered using the CD-HIT-based clustering method [14, 15]. The PyNAST software (http://qiime.org/pynast/) was employed to calculate the number of sequences and operational taxonomic units (OTUs) for each sample by comparing the representative sequence in the Greengene core set database and the NCBI 16SMicrobial database. Then, the species abundance and distribution were analyzed before cluster analysis. Next, sequences were grouped into various OTUs using the Felsentein-corrected similarity matrices, and those within an OTU shared at least 97% similarity [16]. The 16S rDNA was classified into distinct taxonomic categories using the Ribosomal Database Project (RDP) classifier [17–19]. Greengenes 16S rRNA gene database, and NCBI16s (http://home.jmu.edu.cn/xiewn/GenBankhelp.htm) aligned sequences to a curated database of taxonomically annotated sequences. All 16S rDNA sequences were mapped to the RDP database using BLASTN to achieve taxonomic assignments. Sequences that shared greater than 97% identity were used to associate a group of OTUs to specific species, while those less than 97% identity were considered novel reads. Microbial community members were identified through sequence comparison to known bacterial 16S rRNA gene sequences. Alpha diversity (observed species, Shannon, Chaol, and Simpson) and beta diversity (rarefaction curve) were assessed for the impact of the medical intervention on the composition and diversity of the conjunctival microbiota. The bioinformatic analyses of taxonomy (at levels of phylum, class, order, family, genus, and species) and abundance were performed.

### 2.5. Statistical Analyses

All statistical analyses were performed using SPSS19.0 (SPS, Chicago, IL, USA) and GraphPad prism 5.0 (GraphPad Software, San Diego, CA, USA). Alpha index and the relative abundance of the three groups were compared by the one-way analysis of variance, and the difference between any two groups was compared by the Bonferroni *t*-test. The results are expressed as mean ± standard deviation, and a *p* value of <0.05 was considered statistically significant.

## 3. Results

### 3.1. 16S rDNA Sequences from Conjunctival Samples

The 31 patients were 16 men and 15 women, with an average age of 67.5 ± 11.8 years (range, 41 to 84). Sequencing of 93 conjunctival samples from these subjects in three groups generated a total of 2420883 reads corresponding to an average of 26031 gene reads per sample (Table 1). The number of reads for each individual and each group was the basis for comparisons of the number of OTUs. There were 1259 OTUs at 97% sequence similarity, 673 of which were shared by the three groups. The microbial diversity decreased gradually with the administration of 0.5% levofloxacin eyedrops and 5.0% PVI solution. The number of OTUs increased with the number of reads, although no direct proportionality was detected.

### 3.2. Alpha Diversity Analysis

**Figure 1:**
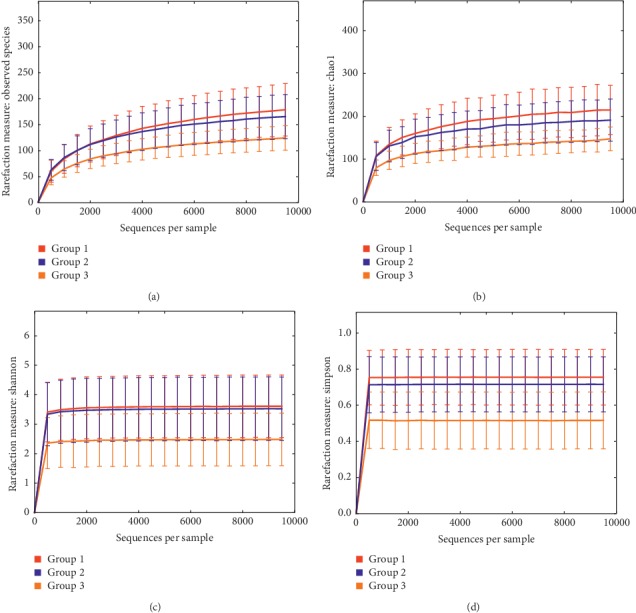
The differences in the indices of observed species, Chao1, Shannon, and Simpson were significant between groups, especially when group 3 was compared with group 1 or group 2. Group 1 was measurably more diverse than group 3. (a) Observed species, treatment. (b) Chao1, treatment. (c) Shannon, treatment. (d) Simpson, treatment.

By assessing alpha diversity, the diversity of microbial communities in the conjunctiva was found to change obviously. The richness and biodiversity of the conjunctival microbiota were evaluated with observed species, Chao1, Simpson index, and Shannon index, respectively (Table 2; Figure 1). Group 1 presented more phylotypes and greater diversity. Similar trajectories were observed in the three groups despite the varied number of reads. Although 0.5% levofloxacin eyedrops altered the richness of the conjunctival bacteria, there was no significant difference between groups 1 and 2. Under interaction of 0.5% levofloxacin eyedrops and 5.0% PVI solution, the richness and biodiversity showed highly significant variability in group 3.

### 3.3. Beta Diversity Analysis

**Figure 2:**
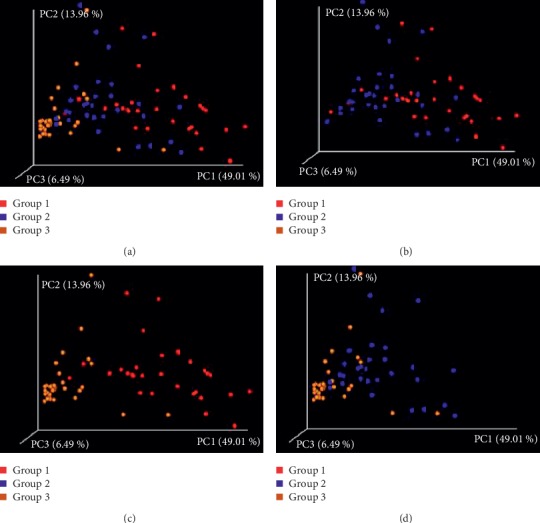
The principal coordinate analysis was derived from weighted UniFrac distances of 16S rRNA gene between microbial communities of the three groups. Values in parentheses on the axis labels indicate that the percentage variation accounts for each axis. (a) The samples were clustered based on different drug interventions. (b) Groups 1 and 2 were a continuum, and there was little distinction between these two groups. (c, d) There was significant difference between group 3 and the other two groups.

Differences between groups were revealed by the analysis of beta diversity. Principal coordinate analysis (PCoA) represents the phylogenetic distance between samples. The beta diversities in the three different groups were calculated and visualized through three dimensional PCoA analyses using the weighted UniFrac distances of 16S rRNA gene between microbial communities (Figure 2). The samples were clustered on the basis of different drug interventions. Significant difference was observed in group 3 compared with the other two groups. Moreover, the samples in group 3 had better consistency mainly due to the use of 5.0% PVI solution.

### 3.4. Bacterial Community Composition

**Figure 3:**
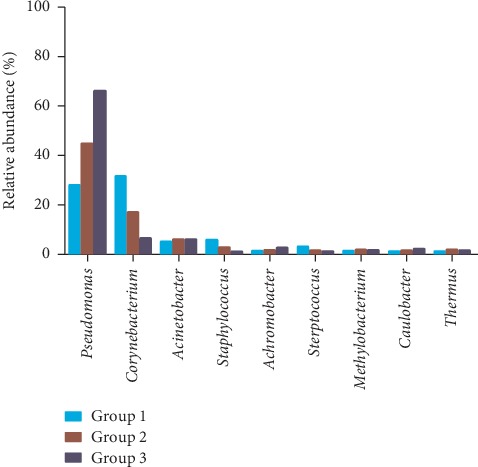
The change tendency of relative abundances of bacterial genera in different groups.

**Figure 4:**
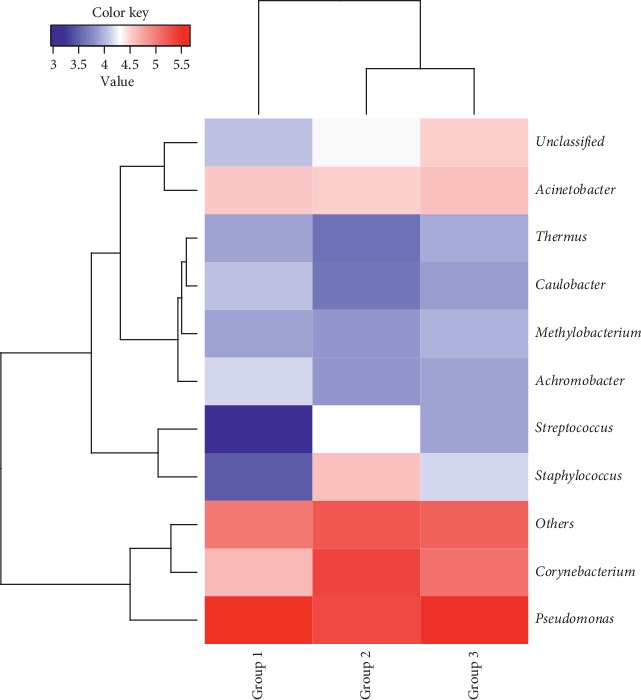
Relative abundances of the top 10 genera in the three groups in a heat map. Values in color key indicated the relative abundance of each genus.

**Figure 5:**
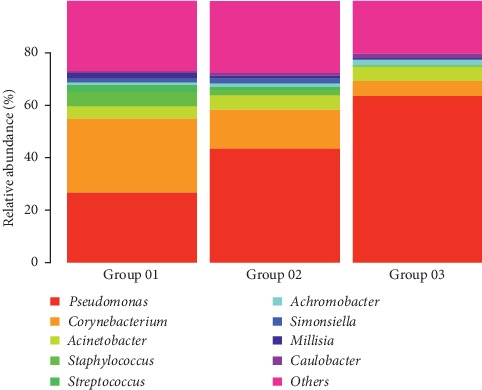
Composition and relative abundances of the conjunctival microbiota in the three groups.

At the 97% confidence threshold in the RDP classifier, the 16S rRNA gene sequencing reads were classified into 25 bacterial phyla, 191 families, and 428 genera in all samples. Despite the nine bacterial genera with the most abundant DNA reads shared among subjects (Figure 3), the relative abundances of the prevalent genera varied significantly, depending on the individual and sample types. The composition of conjunctival bacteria in patients undergoing cataract surgery was obviously altered. At the genus level, the most common bacterial species detected from the conjunctival swabs were *Corynebacterium*, *Pseudomonas*, *Staphylococcus*, *Acinetobacter*, and *Streptococcus* (Figure 4). The susceptibility of different bacterial species to antibiotics and PVI was different. To assess the change in microbial community, the genus relative abundance was compared in pairs (Table 3). After treatment with 5.0% PVI, *Pseudomonas*, *Corynebacterium*, and *Acinetobacter* remained predominant (Figures 4 and 5).

Topical levofloxacin was found to be effective in reducing the quantity and types of bacteria on the conjunctiva. On this basis, the use of 5.0% PVI further strengthened the preoperative conjunctival disinfection. However, bacteria in the conjunctival sac could not be completely removed before surgery.

## 4. Discussion

The rate of positive conjunctival cultures in healthy eyes has been reported to be 60.9% to 100% by using traditional culture-based methods [7, 8]. In our previous study, we found 66.7% (90/135) of positive conjunctival cultures in the prepreparation period [21]. The most commonly encountered organisms in prepreparation cultures were Gram-positive cocci (79/90, with coagulase-negative *Staphylococcus* 94.8%) and *Corynebacterium* (11/90). The eliminating rate of conjunctival bacteria was 72.7% with topical 0.5% levofloxacin, and it increased to 86.4% after adding 5.0% PVI. The distribution of organisms found at baseline in the study was similar to the other reports [7, 8]. Previous studies assessed the efficacy of prophylactic topical antibiotic therapy and PVI use by comparing the number of culture-positive eyes before and after treatments. However, reduction of the total number, composition, and diversity of bacteria seems to be a more appropriate measurement. Meanwhile, culture-based detection is biased toward fast growing bacteria that can be easily cultivated on standard media, so that only a fraction of the microbiota could be observed. It is tricky to detect the rarely encountered, slowly growing, and uncultivable bacteria.

To evaluate the effectiveness of a disinfection method, it is necessary to determine the composition and diversity of bacteria at the surgical area. Because the number of specimens obtained from the area is usually very low, we analyzed the microbial community of the conjunctival sac by the Illumina high-throughput sequencing technology for 16S rDNA. In our previous investigation [11], the RDP classifier was used to classify the 16S rDNA into distinct taxonomic categories by aligning sequences to a curated database of taxonomically annotated sequences for baseline data. In this study, we additionally used the Greengenes 16S rRNA gene database and NCBI16s. Application of the three databases yielded more accurate identification results. It should be emphasized that our data showed a dramatically different prevalence and greater diversity at the genus level than that typically revealed by culture-based methods. Microbiologic surveys of the conjunctival sac performed earlier by our group [21] and others [7, 8] demonstrated a high incidence of *Staphylococcus* and *Corynebacterium*. In contrast, the metagenomic microbial community analysis showed a high prevalence of *Corynebacterium*, *Pseudomonas*, *Staphylococcus*, *Acinetobacter*, and *Streptococcus*, with only 5% of *Staphylococcus*. After topical antibiotic therapy, the composition of conjunctival bacteria in patients was obviously altered, the relative abundance of *Pseudomonas*, *Corynebacterium,* and *Acinetobacter* becoming high, which remained top three after use of 5.0% PVI, indicating that the susceptibility of different bacterial species to antibiotics and PVI varied. The administration of 0.5% levofloxacin eyedrops and 5.0% PVI solution could effectively reduce the amount of bacteria, but they could not completely eliminate the bacteria in the conjunctival sac.

This study also presented information of the relative diversity of the microbial populations according to the beta diversity and alpha diversity results. Rarefaction is ecologically a technique to assess species richness from the results of sampling and allows the calculation of species richness for a certain number of individual samples based on the construction of rarefaction curves. The results of our study demonstrated that the conjunctival bacteria before prophylactic therapy had significantly more phylotypes and greater diversity. In the measurement of diversity, with 0 being equivalent to only one type of microbe in a population and >0 for increasing diversity of the microbial population, the average Shannon index of the baseline conjunctiva was 8.48 and became 8.30 and 8.14 after the application of 0.5% levofloxacin and 5.0% PVI, respectively, indicating that the individuals were colonized by a highly diverse population of microbes, which were reduced slowly with prophylactic therapy. Other measures like Chao1 and Simpson index showed similar results. In a study involving four healthy volunteers, the average Shannon index was 3.09 [9]. A report on the 16S rRNA microbiome from lashes and tears of eyes with (*N* = 7) and without (*N* = 4) blepharitis showed the Shannon index ranging from 0.78 to 3.60, with 81% of samples <3.00 [10]. These differences may be associated with variations in the conjunctival microbiome composition of the enrolled subjects, the method of sample collection, and the processes of trimming and deionizing.

Endophthalmitis prophylaxis remains controversial because of the low incidence of endophthalmitis, and hence there is difficulty in conducting prospective, randomized clinical trials . External ocular floras play an important role in the pathogenesis of acute postoperative endophthalmitis [3–6]. Bacteria of the conjunctival flora mainly consist of *S. epidermidis*, *S. aureus*, *Streptococcus* spp., and other more virulent organisms, such as *Streptococcus pneumonia*, *Diphtheroids*, and *Haemophilus* and *Pseudomonas aeruginosa*, which are the most common causes of postoperative endophthalmitis [22]. Therefore, the goal of preoperative prophylaxis should be reduction of the risk for postoperative endophthalmitis by minimizing the conjunctival bacterial load.

Preoperative antisepsis using PVI is essential for cataract surgery. The American Academy of Ophthalmology ‘‘Cataract in the Adult Eye” Preferred Practice Patterns Guidelines recommend that topical 5.0% PVI eyedrops be instilled into the conjunctival sac preoperatively, whereas the Royal College of Ophthalmology Cataract Surgery Guidelines prefer a flush irrigation of 5.0% PVI into the conjunctival sac [23,24]. The Chinese Ophthalmological Society recommends preoperative prophylactic topical antibiotic therapy for 1 to 3 days and topical 5.0% PVI in conjunctival sterilization for at least 3 minutes [25].

In addition to PVI, the prophylactic topical antibiotic therapy has been extensively performed for the significant reduction of the number of bacteria on the conjunctiva despite the lack of evidence of its effects in reducing postoperative endophthalmitis or the risk for increasing bacterial resistance. Although preoperative administration of topical antibiotics is common, there is no consensus on the combined effectiveness of PVI and antibiotic agents. Some researchers have reported that the combination of topical antibiotics and PVI have greater efficacy in eliminating bacteria compared with either agent alone [26]; others argued that the preoperative bactericidal effect of 5.0% PVI alone in the conjunctival sac was favorable, and there was no significant additive effect by combining it with 0.5% moxifloxacin [27]. In the present study, topical administration of 0.5% levofloxacin significantly reduced the number and changed the composition of conjunctival bacterial flora, and 5.0% PVI could further sterilize the conjunctival sac; however, they could not completely eliminate all bacteria in the conjunctival sac of patients receiving cataract surgery. Therefore, more stringent disinfection methods are needed to control intraoperative aqueous humor contamination caused by bacteria from the conjunctival sac. Researchers have found that intracameral antibiotic injection at the end of surgery or intraoperative irrigation with solutions containing antibiotics significantly reduces the rate of postoperative endophthalmitis [28–30]; however, there is no consensus on the optimal choice of prophylactic routes for prevention of postoperative endophthalmitis; further studies and clinical trials are needed to explore more effective ways to prevent intraocular infections after cataract surgery.

Our study has several limitations. First, the results may be not precisely accurate for evaluation of an effective disinfection method because it is difficult to obtain absolute quantities of microbe expression in the metagenomic analysis. Second, potential sources of error, including sequencing artefacts and taxonomic misidentification, should be involved when short-read next-generation sequencing tools are employed to discover the biodiversity of environmental samples. Third, although we showed that the combination of topical antibiotics and PVI was very effective in eliminating bacteria from the conjunctiva, we did not have data on the efficacy of PVI alone without the use of topical antibiotics. Further investigations are underway. Fourth, the results should be interpreted with caution, as there may be false negatives (from the presence of PCR inhibitors in the sample, bacteria difficult to lyse, or detection threshold) and false positives (from possible contamination at all stages, samples, molecular biology reagents, and handling).

## 5. Conclusion

This study provides a molecular microbial technique for evaluation of the efficacy of a disinfection method. With the metagenomic community analysis of the conjunctival sac using high-throughput techniques, topical 0.5% levofloxacin seems to be effective in reducing conjunctival bacterial flora, and topical 5.0% PVI could make further elimination, but they could not completely eliminate all bacteria in the conjunctiva of patients receiving cataract surgery. More strict disinfection methods need to be adopted to eliminate the bacterial contamination of aqueous humor to further reduce the incidence of endophthalmitis after cataract surgery.

## Figures and Tables

**Table 1:** The number of reads, OTUs, and species in each group.

Group	Reads		OTUs expression	
	Total	Average ± SD	Total	Average ± SD
1	840373	27108.81 ± 6223.94	767401	24754.87 ± 5206.97
2	808865	26092.42 ± 6681.11	740266	23879.55 ± 6114.27
3	771645	24891.77 ± 5657.09	719303	23203.32 ± 4941.69

OTUs, operational taxonomic units (based on 0.03 cutoff) found in each sample; SD, standard deviation.

**Table 2:** The alpha diversity indexes in each group.

	Observed species	Shannon	Simpson	Chao1
Group 1	183.19 ± 52.43	3.617 ± 1.074	0.7558 ± 0.1564	223.71 ± 63.87
Group 2	167.48 ± 44.38	3.524 ± 1.098	0.7158 ± 0.1561	190.93 ± 52.63
Group 3	127.06 ± 25.59	2.480 ± 0.905	0.5168 ± 0.1597	152.13 ± 34.19
Group 1 vs group 2	0.098	0.635	0.217	0.009
Group 1 vs group 3	0.000	0.000	0.000	0.000
Group 2 vs group 3	0.000	0.000	0.000	0.000

Observed species, indicating the number of species (OTU) in the sample; Shannon, computed at the RDP Pyrosequencing Pipeline; Simpson, calculated with MOTHUR [20] using a distance matrix computed at RDP Pyrosequencing Pipeline; Chao1, the estimated richness of an environment based on 0.03 cutoff.

**Table 3:** Relative abundances of the top 5 genera and the pairwise comparison.

	*Pseudomonas*	*Corynebacterium*	*Acinetobacter*	*Staphylococcus*	*Achromobacter*
Group 1	27.48%	30.94%	4.55%	5.26%	0.77%
Group 2	44.10%	16.32%	5.39%	2.19%	1.16%
Group 3	65.55%	5.90%	5.36%	0.39%	2.10%

Group 1 vs group 2	0.000	0.000	0.235	0.010	0.090
Group 1 vs group 3	0.000	0.000	0.997	0.000	0.000
Group 2 vs group 3	0.000	0.000	0.374	0.000	0.000

## Data Availability

All data generated or analyzed during this study are available within the article.
